# Prehabilitation in lung cancer patients undergoing lung resection surgery (Fit4LungNeo): study protocol

**DOI:** 10.1016/j.conctc.2026.101657

**Published:** 2026-06-19

**Authors:** Débora Pedroza Guedes da Silva, Lucimere Bohn, José Oliveira, Ermelinda Eusébio, Maria Gabriela Fernandes, Cristina Teixeira, Hugo Canelas, Pedro Fernandes, Adelino Leite-Moreira, Luísa Gonçalves, Ana Sofia Godinho, João Logarinho Monteiro, Filipa Kendall

**Affiliations:** a Research Centre in Physical Activity, Health and Leisure (CIAFEL) and Laboratory for Integrative and Translational Research in Population Heath (ITR), Faculty of Sport, University of Porto, Rua Plácido da Costa, Porto, 4200-450, Portugal; b Pulmonology Department ULS São João, Alameda Professor Hernâni Monteiro, Porto, 4200-319, Portugal; c Faculty of Medicine, University of Porto, Alameda Professor Hernâni Monteiro, Porto, 4200-319, Portugal; d Nutrition Department, São João, Alameda Professor Hernâni Monteiro, Porto, 4200-319, Portugal; e School of Biotechnology, Universidade Católica Portuguesa (UCP), Rua de Diogo Botelho 1327, Porto, 4169-005, Portugal; f Faculty of Nutrition and Food Sciences, University of Porto, Rua Do Campo Alegre 823, Porto, 4150-180, Portugal; g School of Health Technologies, Polytechnic Institute of Bragança, Campus de Santa Apolônia, Bragança, 5300-253, Portugal; h Department of Cardiothoracic Surgery, ULS São João, Alameda Professor Hernâni Monteiro, Porto, 4200-319, Portugal; i UnIC@RISE, Department of Surgery and Physiology, Faculty of Medicine, University of Porto, Portugal; j School of Health Technologies of Tâmega and Sousa, Polytechnic Health Institute of the North, CESPU, Rua Central de Gandra, 1317, Gandra, 4585-116, Portugal; k H^2^M – Health and Human Movement Unit, School of Health Technologies of Vale Do Ave, Polytechnic Health Institute of the North, CESPU, Rua José António Vidal, 81, Vila Nova de Famalicão, 4760-409, Portugal; l Autonomous Management Unit of Surgery, ULS, São João, Alameda Professor Hernâni Monteiro, Porto, 4200-319, Portugal

**Keywords:** Lung resection, Lung cancer, Prehabilitation, Postoperative pulmonary complications, Length of hospital stay, Exercise training

## Abstract

**Background:**

Lung resection is the *gold standard* and the most effective curative treatment for lung cancer, especially in early-stage non-small cell lung cancer, improving long-term survival and quality of life. The growing body of evidence from randomized controlled trials supports the efficacy of prehabilitation in reducing both the incidence and severity of postoperative pulmonary complications and shortening the length of hospital stay.

**Objective:**

The general objective is to compare the effects of a single education session with different prehabilitation programs in patients undergoing lung resection surgery.

**Methods:**

Two hundred lung cancer patients who are going to be submitted to a lung resection surgery will be randomized into: (1) Inspiratory Muscle Training Group (n = 50), Expiratory Muscle Training Group (n = 50), Global Exercise Training Group (n = 50), and Control Group (n = 50). All patients will receive a single session in-person education session and written information regarding healthy habits to follow before, during, and after hospital discharge, and those from the IMT-G, EMT-G, and GET-G will start their 2-week prehabilitation interventions at ULS São João. Patients will be evaluated at baseline, after completing the prehabilitation program (post-intervention assessment), and 30 days post-discharge (follow-up assessment).

**Discussion:**

Despite diverse intervention strategies, prehabilitation consistently proves effective in bolstering patients' functional capacity. It may also help strengthen the respiratory muscles, which in turn affect lung function and cough effectiveness after thoracic surgery. This randomized trial has been designed to compare different prehabilitation programs and analyze their inherent costs.

## Highlights

• Trial design minimize bias, ensure validity for prehabilitation programs effects.
• Comprehensive patient assessment: objective measures and patient-reported outcomes.
• Supervised prehabilitation programs, adherence and physiological responses.
• Standardized exercise protocols for better understanding postoperative effects.
• Comparison of prehabilitation protocols to identify potentially superior approaches.

## 1 Introduction

Lung cancer is the leading cause of cancer death globally, [1]. In 2022, it accounted for roughly 12.4% of all cancer diagnoses and 18.7% of all cancer-related deaths [2].

Lung resection is the *gold standard* and the most effective treatment for lung cancer, especially in early-stage non-small cell lung cancer (NSCLC), improving long-term survival and quality of life [3]. However, only 15-20% of patients diagnosed with lung cancer are eligible for lung resection [3].

Although the well-known benefits of lung resection, it carries a considerable risk of postoperative pulmonary complications (PPC) occurrence, which negatively impact patients' morbidity and mortality, and escalate hospital costs [4,5]. Previous evidence has suggested that patients with superior functional capacity are better prepared to cope with the stress induced by surgery and, thus, less susceptible to PPCs [6]. The growing body of evidence from randomized controlled trials supports the efficacy of prehabilitation in reducing both the incidence and severity of PPCs and shortening the length of hospital stay (LOS) [[7], [8], [9]].

Prehabilitation lasts the time gap between the decision to proceed with surgery and the surgery. It aims to improve patients’ functional capacity by implementing a multimodal approach that includes medical optimization, physical exercise, nutritional and psychological support [6]. The guidelines for the enhancement of recovery after lung surgery highlight that the preoperative phase should consist of education and counselling, preoperative nutrition, smoking cessation (at least 4 weeks before surgery), alcohol dependency management, anaemia management, and pulmonary rehabilitation and prehabilitation [10].

A meta-analysis encompassing randomized controlled trials showed that patients receiving exercise prehabilitation programs had a lower odds ratio of PPCs (0.31; CI95%: 0.20; 0.48; I^2^ = 0%) and LOS (−3.02; CI95%: −4.82; −1.22; I^2^ = 85%), in comparison with patients who did not receive any intervention [11].

Although there are benefits of prehabilitation on PPCs and LOS, there is a considerable heterogeneity regarding modality, duration, and frequency, making decision-making for health professionals who are in charge of selecting the most successful approach for each patient. While some programs include inspiratory muscle training combined with aerobic exercises, others make different choices, encompassing resistance exercises, educational classes, and incentive spirometry, among many other options [11,12].

The rationale for expiratory muscle training in this study is based on the critical role of these muscles in coughing, airway clearance, and postoperative recovery. Since cough efficacy depends on vigorous expiratory muscle contraction, its impairment directly contributes to PPC [13]. Our previous research showed that patients with lower maximal expiratory pressure (MEP) have an increased risk of PPC (OR 7.44; 95% CI 1.23–19.47) [14]. Furthermore, peak expiratory flow (PEF) is a critical marker of impaired mucociliary clearance, establishing a strong scientific rationale for investigating targeted expiratory muscle training (EMT) as a distinct prehabilitation modality [15].

Based on the existing research gaps, we have designed the Fit4LungNeo project to investigate the effects of different prehabilitation programs in lung cancer patients.

The general objective of this project is to compare the effects of a single education session with different prehabilitation programs in patients undergoing lung resection surgery. Primary objective is to investigate prehabilitation's effectiveness in reducing postoperative pulmonary complications after 30 days of hospital discharge and length of hospital stay, in patients selected for lung cancer resections.

Secondary objectives: i) observe and compare the effect of prehabilitation on pulmonary function, respiratory muscle function, functional capacity, and physical fitness in patients undergoing lung resection surgery; ii) determine the impact of prehabilitation programs on patient-reported outcomes measures (PROMs), including fatigue, dyspnea, anxiety, depression, quality of life, and daily physical activity; iii) determine the cost-effectiveness of each prehabilitation program.

Our primary hypothesis is that all prehabilitation groups will experience a reduction in PPC and LOS, and better functional recovery postoperatively, compared to the usual care control group.

## 2 Material and methods

### 2.1 Design and ethics

The study design and protocol adhere to the Standard Protocol Items: Recommendations for Interventional Trials (SPIRIT) guidelines [16]. Any significant changes to the protocol will be reported to the trial registry and the Ethical Committee. Participation in this study is voluntary and without financial compensation.

Fit4LungNeo is a single-centered, controlled, randomized trial (RCT) with four parallel groups. Two hundred lung cancer patients who are going to be submitted to a lung resection surgery will be randomized into: (1) Inspiratory Muscle Training Group (IMT-G; n = 50), (2) Expiratory Muscle Training Group (EMT-G; n = 50), (3) Global Exercise Training Group (GET-G; n = 50), and (4) Control Group (CG; n = 50).

The study will be conducted by a multidisciplinary team and implemented at the Unidade Local de Saúde de São João (ULS São João) and the Faculty of Sport of the University of Porto (FADEUP) in Portugal.

The Fit4LungNeo was approved by the Ethical Committee from the ULS São João (52/2024) and registered on Clinicaltrials.gov (NCT06802627). All procedures will follow the principles of the Declaration of Helsinki. As it is mandatory in Portugal, civil liability insurance for clinical trials was taken.

### 2.2 Recruitment and screening

The pulmonologist or thoracic surgeon will screen patients for eligibility at the first preoperative appointment at the Cardiothoracic and Pulmonology Departments of the ULS São João. After signing the informed consent, patients will be randomly allocated into one of the four study groups. Those who agree will start the initial assessment and be scheduled for the 2nd assessment day. Recruitment started in June 2025. The inclusion criteria are i. Patients ≥18 years old; ii. diagnosis of lung cancer; ii. Candidates for surgical treatment at the Cardiothoracic Surgery Service of USL São João. The exclusion criteria are i. diagnosis of cardiac or neurological disease, pulmonary hypertension, and renal failure; ii. Previous thoracic surgery; ii. Pneumonectomy; iv. Clinical cognitive and mental disorders; and v. Inability to walk independently; v. Emergency thoracic surgery procedures; vi. Participation in alternative prehabilitation programs.

During the study enrollment phase, we will track the total number of patients screened for eligibility, the number of patients excluded (with reasons for exclusion specified), and the final number of participants randomized [17]. We will report the number of participants who discontinue the intervention, are lost to follow-up, and are included in the intention-to-treat and per-protocol analyses (with reasons).

### 2.3 Randomization

Patient randomization was conducted in Microsoft® Excel, where a list of participants will be generated and the RAND function will be applied to create a column of random numbers. Sorting this column will allow for the random allocation of patients to different study groups.

### 2.4 Blinding

Blinding participants and researchers will not be possible due to the nature of the interventions, except for the researchers responsible for the functional assessment.

### 2.5 Interventions

Regardless of the group allocation, patients will start the intervention one week after the baseline evaluation.

#### 2.5.1 Control group (CG)

Participants from the CG will receive a single in-person education session delivered by a physiotherapist and a nutritionist. The contents of the sessions will be about lifestyle changes regarding nutrition and daily physical activity, abstinence from addictive behaviors, personal hygiene care, and hospital routines. Participants will be encouraged to bring their caregiver or a family member. At the end, participants will be offered a booklet containing all the educational information.

#### 2.5.2 Inspiratory Muscle Training Group (IMT-G)

Participants from the IMT-G will receive the same treatment as the CG, plus 2 weeks of inspiratory muscle training (IMT). The IMT will be 5 days/week, lasting ∼20 min per session, using the POWERBreathe® KH2 device (Warwickshire, England, UK). The sessions will consist of 5 min of stretching exercises followed by IMT. In the first week, the intensity will be set at 50% of the maximal inspiratory pressure (MIP), with eight inspirations per minute. In the second week, there will be an increase of 10% in MIP, with eight inspirations per minute. Patients will be instructed to exercise from residual volume to maximally tolerable inspiratory volume against the inspiratory resistance. Training will be performed seated, wearing a nose clip [18].

#### 2.5.3 Expiratory Muscle Training Group (EMT-G)

Participants from the EMT-G will receive the same treatment as the CG, plus 2 weeks of expiratory muscle training (EMT), 5 days/week, lasting ∼30 min per session. Sessions will start with 5 min of stretching exercises, followed by ∼8 min of specific EMT using the Philips Respironics Threshold PEP® device (Pennsylvania, USA) and conclude with abdominal resistance exercises: curl-ups, curls-ups with trunk rotation and reverse crunch (1st week: 3 exercises, four sets of 8 repetitions; 2nd week: 3 exercises, four sets of 10 repetitions). Patients will be instructed to exercise from total lung capacity until maximal tolerable expiratory volume against the expiratory resistance to the nearby residual volume. Training will be performed seated, wearing a nose clip [18]. The load will be 20 cmH2O, with 6 expirations per minute throughout the intervention.

#### 2.5.4 Global Exercise Training Group (GET-G)

Participants from the GET-G will receive the same treatment as the CG plus 2 weeks of physical exercise, three sessions per week, 45-60 min per session, according to guidelines provided by the American College of Sports Medicine [19]. The GET sessions will include a warm-up, main workout, and cooldown. The warm-up and cool-down will last ∼5 min each and will encompass whole-body stretching exercises, holding the point of feeling light discomfort for 15 s. The main workout will have an aerobic and a resistance component. Aerobic: In the first week, participants will walk for 20 min on a treadmill at 70% of the mean speed achieved on the 6-min walk test and be limited by 80% of the maximum theoretical heart rate. In the second week, the intensity will increase to 80% of the mean speed achieved on the 6-min walk test but will still be limited by 80% of the maximum theoretical heart rate. Resistance: Resistance training using body weight and elastic bands will consist of 8 exercises (squats, wall chest press, calf raises, low-row, biceps plus shoulder press, curl-up, curl-up, curl-up with trunk rotation and reverse crunch). In week 1, participants will perform two sets of 8-10 repetitions, progressing to two sets of 10-12 repetitions in week 2.

Patients in exercise groups will be continuously monitored by an oximeter and self-perceived effort scale (0-10). Exercise sessions for all groups will be conducted and supervised by a physiotherapist at ULS São João.

### 2.6 Evaluation moments

**Fig. 1:**
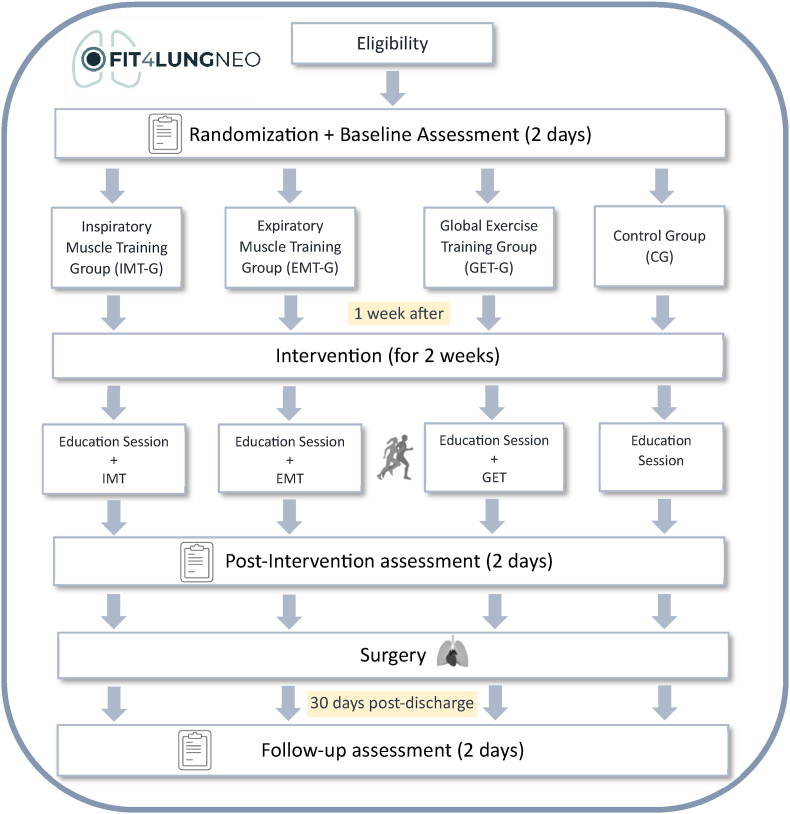
Study design and timeline Flowchart of study procedures. IMT: Inspiratory Muscle Training; EMT: Expiratory Muscle Training; GET: Global Exercise Training Group.

Patients will be evaluated at baseline, after completing the prehabilitation program (post-intervention assessment), and 30 days post-discharge (follow-up assessment) (Fig. 1). In each one of the assessments, 2 days of evaluation will be needed to complete all the procedures.

In brief, on the first day of assessments, a physician will screen for eligibility criteria and forward the eligible patients to a physiotherapist and a nutritionist, who will collect data on anthropometry, body composition, sociodemographic, clinical history, and behavioral risk factors. Thereafter, questionnaires regarding PROMs will be applied in the form of interviews. Finally, patients will be provided with an accelerometer to measure daily physical activity during waking hours until the completion of the prehabilitation program.

Patients will be evaluated for pulmonary function, respiratory muscle function, functional capacity, and physical fitness on the second assessment day.

Afterward, all patients will receive an education class and written information regarding healthy habits to follow before, during, and after hospital discharge, and those from the IMT-G, EMT-G, and GET-G will start their 2-week prehabilitation interventions at ULS São João. The study design and timeline are presented in Fig. 1.

After the prehabilitation programs, all patients will be reassessed using the same baseline procedures and then undergo the surgery. The surgical team will register information on ASA classification, ARISCAT score, minimum oxygen saturation, surgical approach, total surgical time, and extent of resection. After surgery, all patients will receive standard care, including daily medical assessment, physiotherapy care, and analgesic protocol.

PPC and other complications and LOS will be documented based on the patient's medical record. Any postoperative complications and hospital readmissions within the following 30 days will also be monitored and recorded.

After hospital discharge, patients will be referred to outpatient physiotherapy and nursing care. Three weeks post-discharge, patients will attend a medical appointment and will be scheduled for the follow-up assessment. Participants will receive an accelerometer to wear for one week. Upon completion of data collection, a cost-effectiveness analysis will be conducted to determine the actual economic cost-benefit of each protocol.

### 2.7 Attendance

Attendance will be defined as the percentage of sessions attended by the patient (recorded by the physiotherapists) divided by the total number of sessions (GET-G: 6 sessions, IMT-G and EMT-G: 10 sessions).

### 2.8 Adverse events and risks

The occurrence and causes of adverse events will be monitored, recorded, and reported separately for each study group.

### 2.9 Outcomes

The following subsections delineate all the outcomes (summarized in Table 1) and explain how the researchers will measure them.

**Table 1:** Outcomes instruments and time points of data collection.

Outcomes	Instruments	Time Points		
Primary Outcomes		Baseline	Post-intervention	Follow up
Postoperative pulmonary complications (PPC)	occurrence of postoperative pulmonary complications from surgery to 30 days after discharge according to the ESTS and STS (Fernandez et al., 2015).			x
Length of hospital stay (LOS)	number of days of postoperative hospitalization including hospital readmissions related to surgery, assessed in clinical data.			x
**Secondary Outcomes**		Baseline	Post-intervention	Follow up
Pulmonary function	informations captured using the equipment Quark PFT®, Cosmed (Rome, Italy).	x	x	x
Respiratory muscle function				
Aerobic endurance	6-min walk test (6-MWT), according to the American Thoracic Society (ATS) guidelines (2002).	x	x	x
Lower body strength	sit-to-stand test of the Senior Fitness Test (JONES, RIKLI & BEAM, 1999).	x	x	x
Handgrip strength	dynamometer Jamar Plus + Digital hand dynamometer (Sammons Preston Inc., Bolingbrook, Illinois, USA).	x	x	x
Balance	balance test, speed walk test (4 m) and stand-up and sit-down test (5 times) from the Short Physical Performance Battery (SPPB) (GURALNIK et al., 1994).	x	x	x
Speed walk				
Lower body power				
agility, coordination and dynamic balance	time up and go test (TUG) (Podsiadlo et al., 1991).	x	x	x
Dyspnea	modified Medical Research Council (mMRC) Dyspnea Questionnaire (RIBEIRO et al., 2022).	x	x	x
Fatigue	Fatigue Assessment Scale (FAS) (MICHIELSEN et al., 2004).	x	x	x
Anxiety and depression	Hospital Anxiety and Depression Scale (HADS) (PAIS-RIBEIRO et al., 2007).	x	x	x
Quality of life	EuroQol 5D-5L (FENG et al., 2021).	x	x	x
subjective daily physical activity	Physical Activity Questionnaire - Short Form (IPAQ-SF) (CRAIG et al., 2003).	x	x	x
objectively daily physical activity	GT3X + accelerometers (ActiGraph®, LLC, Pensacola, FL, USA).	x	x	x
Economic impact	Specific costs attributed to each intervention and comparison cost-effectiveness of each program.			x
**Other Outcomes**		Baseline	Post-intervention	Follow up
Sociodemographic variables	Interview.	x		
Medical history				
Barriers for prehabilitation program	Barriers questionnaire for prehabilitation (adapted from GHISI et al., 2012).	x		
Body Composition	bioimpedance (InBody 770®, InBody Co. Ltd., Seoul, South Korea).	x	x	x
Body mass index	Scale InBody 770 ®, InBody Co. Ltd., Seoul, South Korea)	x	x	x
tabaco smoking habits	Calculated by formula pack-years (BERNAARDS et al., 2001).	x	x	x
Food intake	1-day food record.	x	x	x

Post-intervention assessments (after finishing 2 weeks of intervention) and follow-up assessments (after 30 days of hospital discharge).

### 2.10 Primary outcomes

PPC (from surgery to 30 days after discharge) will be screened according to the joint agreement and standardized definitions provided by the European Society of Thoracic Surgery (ESTS) and the Society of Thoracic Surgeons (STS) [20].

LOS and readmissions (from surgery to 30 days after discharge) will be based on the number of postoperative hospitalization days, including hospital readmissions related to surgery, assessed in clinical data. For those patients with persistent air leak, allowed to return home with a drainage tube, it be resisted the number of days util remove the tube.

### 2.11 Secondary outcomes

Except for the economic cost-effectiveness analysis, all secondary outcomes will be assessed at baseline, post-intervention (before surgery), and follow-up (30 days after hospital discharge).

#### 2.11.1 Pulmonary and respiratory muscle function

Pulmonary function will be assessed through absolute and relative values of forced vital capacity (L) and (%); forced expiratory volume in 1 s (L) and (%); peak expiratory flow (L/s) and (%); Tiffeneau Index (%); total lung capacity (L) and (%); diffusion lung capacity for carbon monoxide (mmol/min/kPa) and (%); diffusion lung capacity for carbon monoxide per unit alveolar volume (mmol/min/kPa/L) and (%) [21].

Respiratory muscle function will be measured through absolute and relative values of maximal voluntary ventilation (L/min) and (%); maximal inspiratory pressure (cmH2O) and (%); maximal expiratory pressure (cmH2O) and (%) [22].

This information will be captured using the equipment Quark PFT®, Cosmed (Rome, Italy).

#### 2.11.2 Functional capacity and physical fitness

Functional capacity and physical fitness will be evaluated through changes in aerobic endurance, lower body strength, handgrip strength, balance, speed walk, lower body power, agility, coordination, and dynamic balance, as described below.

Aerobic endurance will be assessed by the 6-min walk test (6-MWT), according to the American Thoracic Society (ATS) guidelines [23]. Distance (in meters), maximum and minimum oxygen saturation (%), and maximum and minimum heart rate (bpm) will be recorded. Both oxygen saturation and heart rate will be collected using the Spirodoc® (Rome, Italy).

Lower body strength changes will be measured using the sit-to-stand test of the Senior Fitness Test [24]. Handgrip strength will be assessed using the dynamometer Jamar Plus + Digital hand dynamometer (Sammons Preston Inc., Bolingbrook, Illinois, USA). Measurements will be carried out according to the American Society of Hand Therapists’ Recommendations [25].

The Short Physical Performance Battery (SPPB) will be used to assess balance, walking speed, and lower body power [26,27]. Agility, coordination, and dynamic balance will be measured by the timed up and go test (TUG) [28].

#### 2.11.3 Patient-reported outcome measures (PROMS)

Dyspnea will be evaluated using the modified Medical Research Council Dyspnea Questionnaire (mMRC) [29]. Fatigue will be determined through the Fatigue Assessment Scale (FAS) [30]. Anxiety and depression will be evaluated with the Hospital Anxiety and Depression Scale (HADS) [31].

Quality of life will be assessed with the EuroQol 5D-5L. This questionnaire contains the dimensions of mobility, self-care, usual activities, pain/discomfort, anxiety/depression, and participants also classified their perception of general health status [32].

#### 2.11.4 Daily physical activity

Daily physical activity will be objectively and self-reportedly assessed. The International Physical Activity Questionnaire - Short Form (IPAQ-SF) will assess self-reported information about the weekly volume spent in vigorous, moderate, and light physical activity and the time spent sitting [33].

Additionally, participants will wear GT3X + accelerometers (ActiGraph®, LLC, Pensacola, FL, USA) along with waking hours over their right hip during 7 days at the beginning of the study, during the 2-week intervention period, and for 7 days at the follow-up assessment. Accelerometer data will be processed using ActiLife software (ActiLife®, LLC, Pensacola, FL, USA).

#### 2.11.5 Economic impact

Specific costs are attributed to each intervention, and the cost-effectiveness of each program is compared, considering the occurrence and severity of PPC, other complications, LOS, and hospital readmissions related to surgery.

### 2.12 Other outcome measures

This section outlines additional outcome measures that will be collected to provide a comprehensive understanding of the study participants and potential influences on the primary and secondary outcomes.

At baseline, participants will undergo an interview to assess their sociodemographic variables such as age (years), academic level, and civil status. The medical history, including any diagnosed diseases and the number of medications they are currently taking, will be documented by one of the research members.

Furthermore, potential barriers for the prehabilitation program will be evaluated at baseline using a questionnaire adapted from Ghisi et al. (2012) [34]. All patients who fulfill the inclusion criteria, even those who decline to participate in the clinical trial, will be asked to answer the questionnaire. This 21-item questionnaire employs a 5-point response scale for the initial 20 questions and allows for an open-ended response to the final question.

Body composition (body mass (kg), fat-free mass (kg), and fat mass (kg)) will be measured at three assessment points using bioimpedance analysis with the InBody 770® device (InBody Co. Ltd., Seoul, South Korea). Body Mass Index (BMI) will also be calculated.

Tobacco smoking habits will also be recorded at three assessment points categorized as “never smoker”, “former smoker”, or “smoker”. The tobacco consumption will be calculated by the number of pack-years using the formula: number of cigarettes per day/20 x number of smoking years [35].

Finally, food intake, including alcohol consumption, will be measured using a 1-day food record, for which participants will receive detailed instructions on accurate recording. The collected dietary data will then be analyzed using the Food Processor Software®.

### 2.13 Sample size

To accomplish the aim of this project it was calculated the sample size based on the effect size reported by Cavalheri and Granger (2017) [36] of preoperative exercise training in pulmonary complications after lung resections (0.33) and considering an α = 0.05 and a statistical power of 95%, the required sample is 180 patients, 45 for each group (G∗Power version 3.1.9.2; Universität Düsseldorf, Germany) [37]. However, assuming a percentage of dropouts around 10%, our study's estimated total sample size will be 200 patients (50 in each study arm).

### 2.14 Statistical analysis

A framework for testing superiority will be applied. In the primary analyses, we will compare the effectiveness of both exercise interventions to usual care (no exercise training). Final analyses will be conducted after the collection and processing of data for the primary and secondary outcomes. The time points for data collection of each outcome are outlined in the outcomes section.

### 2.15 Data management and sharing

After signing the informed consent form, each participant will be assigned a unique code number to safeguard their data and minimize the risk of unauthorized access or disclosure. Any information that links the pseudonym to identifiable details will be stored separately and managed to ensure it cannot be traced back to any individual.

The study will prioritize governance, ethical considerations, and comprehensive trial oversight. The protocol, plan for statistical analyses, and data management strategy will be openly accessible. Whether positive, negative, or inconclusive, results will be disseminated through peer-reviewed journals and presented at national and international conferences. The collected data will undergo double verification by a research team member.

## 3 Discussion

Despite differing intervention strategies, prehabilitation consistently proves effective in bolstering patients' functional capacity [38]. Given that systemic inflammation and surgical stress commonly lead to reduced postoperative functional capacity—a major predictor of morbidity and mortality after lung surgery—optimizing physiological reserves preoperatively is crucial [39].

This need for enhanced physiological reserves is particularly relevant given that physical inactivity presents a significant global health challenge, disproportionately affecting older adults and leading to rapid deterioration of aerobic capacity and functional fitness and increased susceptibility to chronic illnesses. This inactivity can amplify the risk of post-surgical complications, particularly during major procedures [40]. Fortunately, studies demonstrate that preoperative exercise programs are effective in improving exercise capacity among lung cancer patients scheduled for surgical resection [41].

A recent systematic review and meta-analysis found that cancer patients with higher muscle strength or cardiorespiratory fitness levels had a significantly lower risk of all-cause mortality compared to those with lower physical fitness levels. Additionally, muscle strength and cardiorespiratory fitness were strong predictors of mortality, especially in patients with advanced cancer, and physical fitness components were also linked to a reduced mortality risk in lung and digestive cancers [42].

Prehabilitation may also help strengthen the respiratory muscles that affect functional capacity lung function, and cough effectiveness after thoracic surgery, consequently improving functional capacity [39].

Regarding the value of inspiratory muscle training, especially when applied in the preoperative period and pulmonary surgery, it was shown in a meta-analysis that it is effective in reducing PPC and LOS in patients undergoing surgery [43].

Evidence suggests that prehabilitation offers potential economic advantages by reducing complications, shorter hospital stays, and better patient outcomes. Assessing the economic impact of integrating prehabilitation programs, alongside their effectiveness for lung cancer patients, requires a comprehensive evaluation of the costs involved in improving care quality and the costs associated with program implementation [38]. This includes treatment expenses and the costs borne by healthcare providers [38], because effective prehabilitation programs require a multidisciplinary approach, integrating expertise from various healthcare professionals across different sectors [9].

The clinical implications of improving respiratory muscle function extend beyond the prevention of PPC. Our study seeks to validate how targeted prehabilitation can directly impact the use of hospital resources, given that previous data indicate that the combination of MIP%, MEP, and TLC% can explain 59% of the variation in LOS [44].

By comparing different training programs, our study aims to identify which intervention strategies can lead to more effective prehabilitation strategies, resulting in cost-effective and personalized treatments for lung cancer patients.

Although the effectiveness of prehabilitation programs is increasingly documented, there is a relative scarcity of studies investigating the costs associated with these interventions [45].

Based on these findings, our study will analyze the effects of the applied prehabilitation programs and their inherent costs.

### 3.1 Strengths and limitations

This study possesses several notable strengths that enhance the reliability and validity of its findings. Firstly, the RCT design ensures rigorous participant allocation, minimizing selection bias and bolstering the validity of causal inferences. Secondly, the comprehensive assessment of functional capacity provides a holistic view of patient health. Thirdly, including PROMs, captures the patient's subjective experience, providing a well-rounded understanding of the intervention's impact. Fourthly, monitoring heart rate and oxygen saturation during exercise sessions allows for detailed adherence and physiological response analysis. Fifthly, the standardized pre- and post-operative care protocols and controlled warm-up, cool-down, and exercise program duration strengthen the attribution of observed effects to the exercise intervention. The study's mechanistic approach offers insights into how lifestyle and daily habit changes influence patient health and whether the intervention is sufficient to elicit positive post-operative changes. Finally, this study addresses a clinically relevant question, specifically evaluating the comparative effectiveness of different prehabilitation modalities and identifying potential superior interventions.

The study has some limitations that need to be considered. Although it may influence the results, including patients in neoadjuvant therapy will be controlled by statistical analysis. Risk stratification, in turn, will be used as an analysis variable, and not as an exclusion criterion. The inclusion of only individuals without heart disease, although it may limit the number of participants, aims to isolate the effect of the intervention on pulmonary complications, minimizing the influence of comorbidities. The relatively short duration of the intervention (2 weeks), although it may be sufficient for physiological changes, may not be ideal for long-term adaptations. However, it is important to remember that this period was defined so there would be no delays in the patient's surgery. The absence of blinding, justified by the nature of the intervention, will be mitigated by standardizing procedures and using independent evaluators. These limitations, however, are balanced by the strengths of the study.

## Funding source

This work was supported by 10.13039/501100001871FCT– Fundação para a Ciência e a Tecnologia, I.P., under project 2023.16437.ICDT, DOI identifier https://doi.org/10.54499/2023.16437.ICDT. FCT supported Débora Pedroza Guedes da SIlva with a doctoral grant 2023.01704.BD, DOI identifier https://doi.org/10.54499/2023.01704.BD and the researcher Lucimere Bohn (CEECINSTLA/00007/2022/CP2914/CT0003). DOI identifier https://doi.org/10.54499/CEECINSTLA/00007/2022/CP2914/CT0003.

The Research Center in Physical Activity, Health and Leisure (10.13039/501100018710CIAFEL), Faculty of Sport, 10.13039/501100006752University of Porto (FADEUP), and the Laboratory for 10.13039/100018696Integrative and Translational Research in Population Health (ITR) are funded by the Portuguese Fundação para a Ciência e Tecnologia(FCT) grants UID/00617/2025: https://doi.org/10.54499/UID/00617/2025 and LA/P/0064/2020 https://doi.org/10.54499/LA/P/0064/2020.

## CRediT authorship contribution statement

**Débora Pedroza Guedes da Silva:** Conceptualization, Methodology, Writing – original draft, Writing – review & editing. **Lucimere Bohn:** Conceptualization, Methodology, Project administration, Validation, Writing – original draft, Writing – review & editing. **José Oliveira:** Conceptualization, Methodology, Supervision, Writing – review & editing. **Ermelinda Eusébio:** Conceptualization, Methodology, Writing – review & editing. **Maria Gabriela Fernandes:** Conceptualization, Methodology, Project administration, Writing – review & editing. **Cristina Teixeira:** Conceptualization, Methodology. **Hugo Canelas:** Methodology, Software, Writing – review & editing. **Pedro Fernandes:** Conceptualization, Methodology. **Adelino Leite-Moreira:** Conceptualization, Methodology. **Luísa Gonçalves:** Methodology, Writing – review & editing. **Ana Sofia Godinho:** Conceptualization, Methodology, Writing – review & editing. **João Logarinho Monteiro:** Conceptualization, Methodology. **Filipa Kendall:** Conceptualization, Funding acquisition, Methodology, Project administration, Supervision, Writing – original draft, Writing – review & editing.

## Declaration of competing interest

The authors declare the following financial interests/personal relationships which may be considered as potential competing interests:Debora Silva reports financial support was provided by Foundation for Science and Technology. Ermelinda Eusebio, Maria Gabriela Fernandes, Cristina Teixeira, Hugo Canelas, Pedro Fernandes, Adelino Leite-Moreira, Ana Sofia Godinho, Joao Logarinho Monteiro, Filipa Kendall reports a relationship with University Hospital Center of Sao Joao that includes: employment. If there are other authors, they declare that they have no known competing financial interests or personal relationships that could have appeared to influence the work reported in this paper.

## Data Availability

No data was used for the research described in the article.
